# Artificial intelligence *versus* traditional approaches in multicomponent spectral analysis

**DOI:** 10.1038/s41598-026-39433-3

**Published:** 2026-03-01

**Authors:** Nesma M. Fahmy, Reem H. Obaydo, Hayam M. Lotfy

**Affiliations:** 1 Faculty of Pharmacy, Pharmaceutical Chemistry Department, Ahram Canadian University, Giza, Egypt; 2 Faculty of Pharmacy, Department of Analytical and Food Chemistry, Ebla Private University, 22743 Idlib, Syria; 3 Faculty of Pharmacy, Pharmaceutical Analytical Chemistry Department, Cairo University, El-Kasr El-Aini Street, Cairo, 11562 Egypt

**Keywords:** Tolnaftate, Artificial intelligence, Betamethasone, Double divisor derivative ratio, Factorized spectrum, Smart analytical chemistry, Chemistry, Computational biology and bioinformatics, Mathematics and computing

## Abstract

This study explores the use of AI-assisted data handlingin spectrophotometric method development, providing a flexible and globally accessible alternative to traditional manual software algorithms.Quadriderm cream combines four active ingredients: Clioquinol (CLIO), Betamethasone (BETA), Tolnaftate (TOL), and Gentamicin (GEN) with the preservative Chlorocresol (CC). Building on our previous research on complex pharmaceutical mixtures with challenging ratios, this study applied established protocols for CLIO and GEN while focusing on the more analytically demanding ternary subsystem (TOL, BETA, and CC).The integration of AI-enhanced spectral handling and interpretation reduces operator-dependent variability and streamlines the analytical workflow. This includes generating calibration graphs and regression equations, as well as effectively handling scanned spectral data via consecutive prompts. Validation data such as accuracy and precision are assessed to ensure reliability. Furthermore, the system enables intelligent, simultaneous analysis of laboratory mixtures and pharmaceutical formulations, enhancing both efficiency and accuracy. The AI strategy, trained on spectral data supplied and monitored by the expertiseanalyst, can automatically predict optimal wavelengths with minimal interference, while manual handling strategy rely on analyst-driven selection. Two novel approaches were developed: the factorized derivative ratio extraction using double divisor (MAN-[DD- DDE])via Spectra Manager^®^ software and the automated double divisor derivative ratio (AUTO-[DD-DD]) via AI tools and for resolving ternary mixtures with severely overlapping UV spectra and comparing the results with those of(MAN-[DD- DD])at coincidence points. Linear working ranges were 0.5–5.0 µg/mL (TOL), 3.0–30.0 µg/mL (BETA), and 2.0–20.0 µg/mL (CC); LODs were 0.09, 0.09, and 0.26 µg/mL, respectively. AI-driven data processing strategy matched the accuracy and reproducibility of traditional strategy manipulation while reducing subjective steps and effort. Finally, the UV-spectrophotometric method for pharmaceutical cream analysis was evaluated using the MA Tool (2025) to assess sustainability across green, white, and AI-driven criteria. AI-assisted scoring via Microsoft Copilot enabled rapid, reproducible assessment, yielding a Whiteness Score of 60.9% and providing actionable recommendations for greener and more efficient workflows.

**Supplementary Information:**

The online version contains supplementary material available at 10.1038/s41598-026-39433-3.

## Introduction

UV–Vis spectrophotometry remains a cornerstone of pharmaceutical analysis due to its simplicity, low cost, and non-destructive nature; however, selectivity becomes challenging in multicomponent systems with extensive spectral overlap, increasing reliance on preprocessing strategies and wavelength selection. Recent advances in artificial intelligence (AI) provide a complementary solution through enhanced pattern recognition and feature extraction, enabling rapid screening of preprocessing routes and analytical wavelengths while preserving the chemist’s role in theoretical validation and performance assessment^1–5^. AI-based approaches, including machine learning, neural networks, and automated data-processing workflows, have demonstrated strong capabilities in modeling complex spectral data, minimizing noise and interferences, and improving quantification and selectivity even in poorly resolved systems^6–8^. By enabling prompt-driven automation of key analytical steps such as baseline correction, feature extraction, and peak resolution, AI enhances efficiency and reproducibility without replacing human expertise. Unlike traditional spectrophotometer software (e.g., Spectra Manager^®^), which depends on sequential manual operations that are time-consuming and error-prone, AI-assisted workflows process complete spectral datasets directly from CSV files using analyst-guided structured prompts. Systematic comparison with conventional software confirms the reliability and efficiency of AI-assisted spectrophotometric data handling, underscoring its value as an intelligent support tool for advanced analytical workflows.

Quadriderm^®^ cream contains Clioquinol (CLIO), Betamethasone (BETA), Tolnaftate (TOL), and Gentamicin (GEN), with Chlorocresol (CC) as a preservative. CLIO is a broad-spectrum antimicrobial and antifungal agent^9^, BETA is a potent corticosteroid with anti-inflammatory and antipruritic activity, TOL is an antifungal effective against dermatophytes^10^, and GEN provides antibacterial coverage against Gram-negative organisms^11^. The combination ensures simultaneous control of infection and inflammation, optimizing therapeutic efficacy in dermatological conditions.

Previous studies have analyzed components of Quadriderm^®^ cream individually or in partial mixtures. HPLC methods quantified CLIO and BETA in the presence of GEN and TOL^12^ and LC-MS/MS methods determined CLIO, TOL, and BETA with GEN and CC as interferences^13^.

This study builds on our previous work^1^ on complex mixtures of CLIO, BETA, TOL, GEN and CC with challenging ratios by extending established protocols for CLIO and GEN to a more demanding ternary system of TOL, BETA, and CC, which serves as a good model for developing and comparing spectrophotometric methods using both conventional software and AI-driven platforms. Evaluating these parallel strategies enables assessment of resolution efficiency, operational simplicity, traceability, and robustness, highlighting the adaptability and reliability of AI-assisted method development, compared to conventional methods and those previously applied in the literature^1^. A central aim is to establish AI as a universal platform (UP) that enables laboratories in resource-constrained settings to perform advanced method development without dependence on licensed software. Unlike proprietary platforms with rigid workflows, training requirements, and legal restrictions, free AI tools such as ChatGPT and Microsoft Copilot offer accessible, modifiable, and shareable solutions that support automated data processing, predictive modeling, and real-time optimization while reducing cost and legal risk^7,8^.

Accordingly, this work develops and validates eco-friendly UV–Vis spectrophotometric methods based on derivative ratio techniques for simultaneous analysis of the resolved ternary mixture, with data processing performed either manually using Spectra Manager^®^ or via AI-assisted workflows. Comparative evaluation demonstrates the ability of AI, under analyst guidance, to predict spectral regions, execute resolution steps, and enhance reproducibility. Additionally, advanced double divisor approaches, including DD-DDE, are assessed against the conventional DD-DD method. Finally, method sustainability is evaluated using the MA Tool (2025), which integrates environmental impact, applicability, analytical performance, and innovation domains, supporting green and white analytical chemistry principles while improving the transparency, efficiency, and reproducibility of sustainability assessment through AI-assisted documentation and visualization^14,15^.

### Theoretical background

#### Manual double divisor-derivative ratio extraction method [MAN-DD-DDE]

The DD-DDE method combines mathematical calculations and in-silico manipulation to accurately extract the derivative ratio spectrum of Z from a ternary mixture (X, Y, and Z). This approach offers improved accuracy and robustness compared to traditional DD-DD methods, which rely on a critical coincidence point measurement. By leveraging peak-to-peak measurement, DD-DDE allows for maximum sensitivity. The method involves coupling the factorized spectrum with the peak amplitude value of the derivative ratio spectra (DD) of the ternary mixture, using an equimolar mixture of two interfering components as a divisor^1–3,16^.

1 $$\:=\:\frac{\mathrm{a}\mathrm{x}\:\mathrm{C}\mathrm{x}}{\:\mathrm{a}\mathrm{x}\mathrm{C}\mathrm{x}\:+\:\mathrm{a}\mathrm{y}\mathrm{C}\mathrm{y}}+\frac{\mathrm{a}\mathrm{y}\mathrm{C}\mathrm{y}}{\:\:\mathrm{a}\mathrm{x}\mathrm{C}\mathrm{x}\:+\:\mathrm{a}\mathrm{y}\mathrm{C}\mathrm{y}\:}+\frac{\mathrm{a}\mathrm{z}\mathrm{C}\mathrm{z}}{\:\:\mathrm{a}\mathrm{x}\mathrm{C}\mathrm{x}\:+\:\mathrm{a}\mathrm{y}\mathrm{C}\mathrm{y}}$$

where ax, ay, az and Cx, Cy and Czresemble the absorptivitiesand the concentrations of X, Y, and Z.

Where Cx = Cy

2 $$=\frac{\mathrm{a}\mathrm{z}\mathrm{C}\mathrm{z}}{\mathrm{C}\mathrm{x}\:\left(\mathrm{a}\mathrm{x}+\:\mathrm{a}\mathrm{y}\right)^{\prime}}+\left[\frac{\left(\mathrm{a}\mathrm{x}\mathrm{C}\mathrm{x}\:+\mathrm{a}\mathrm{y}\mathrm{C}\mathrm{y}\right)}{\mathrm{C}\mathrm{x}\:\left(\mathrm{a}\mathrm{x}+\mathrm{a}\mathrm{y}\right)^{\prime}}\right]$$

The study used normalized spectra of interfering components (X + Y), each at 1.0 µg/mL, in a double divisor representing the sum of their absorptivity spectra [ax + ay]. These normalized spectra, averaged from multiple concentrations, offer advantages over concentration-dependent divisors by reducing errors from sample preparation, dilution, instrumental variability, and concentration inaccuracies. This approach enhances response accuracy, simplifies use without requiring precise standard solutions, and minimizes noise amplification while maintaining sensitivity. At certain point of wavelength, the ratio of mixtures is the sum of $\:\:\mathrm{a}\mathrm{x}\mathrm{C}\mathrm{x}\:+\mathrm{a}\mathrm{y}\mathrm{C}\mathrm{y}$ to [$\:\mathrm{a}\mathrm{x}\:+\mathrm{a}\mathrm{y}]^{\prime}$ is equal to a constant (k) with respect to λ .

3 $$=\frac{\mathrm{a}\mathrm{z}\mathrm{C}\mathrm{z}}{\left(\mathrm{a}\mathrm{x}+\:\mathrm{a}\mathrm{y}\right)^{\prime}}+\mathrm{k}$$

First derivative of Eq. (3) is calculated using Δ λ = 4 and scaling factor 10, knowing that the derivative of a constant is zero at certain point (coincidence point), Eq. (4) would beacquired:

4 $$=\frac{\mathrm{d}}{\mathrm{d}{\uplambda\:}}\:\left\{\frac{\mathrm{a}\mathrm{z}}{\left(\mathrm{a}\mathrm{x}+\:\mathrm{a}\mathrm{y}\right)^{\prime}}\right\}\left\{\mathrm{C}\mathrm{z}\right\}$$

Equation (4) serves as the mathematical basis for analyzing component Z via [MAN-DD-DD],

the derivative signal of a ternary mixture’s ratio spectrum shows a dependence on concentrations of Z (C_Z_ ) but is independent of concentrations C_X_ and C_Y_. To practically identify the coincidence point (CP), the first derivative of the ratio spectra of the ternary mixture and pure Z standard are overlaid, both using normalized spectra of X + Y .

For [MAN-DD-DDE] method, factorized double divisor derivative ratio spectrum of Z [FDD-DD-S] is introduced as resolving spectrum in [DD-DDE] method. This spectrum is obtained by division of the derivative ratio spectrum of a certain concentration of pure Z (within its linearity range) using the sum of normalized spectra of X and Y as a divisor by the values of peak amplitude ($\mathrm{P}\left({\lambda}\mathrm{cp}\right)$ at the (CP).

5 $$\mathrm{MAN-FDD-DD-S.}=\:\frac{d}{d\lambda\:}\left\{\frac{\mathrm{a}\mathrm{z}}{\left(\mathrm{a}\mathrm{x}+\mathrm{a}\mathrm{y}\right)^{\prime}}\right\}\left\{\mathrm{C}\mathrm{z}\right\}/\mathrm{P}^{\prime}_{\left({\lambda }\mathrm{CP}\right)}$$

To get the derivative ratio spectrum of Z in the ternary mixture; multiplication of amplitude value of each mixture at the specified coincidence wavelength ($\mathrm{P}_{\left(\lambda\mathrm{CP}\right)}$) by the factorized spectra {FDD-DD-S} of Z.

$\:\therefore\:$Recovered double divisor derivative ratio of Z

6 $$=\frac{d}{d\lambda}\left\{\frac{\mathrm{a}\mathrm{z}}{\left(\mathrm{ax}+\mathrm{ay}\right)^{\prime}}\right\}\left\{\mathrm{C}\mathrm{z}\right\}=\left[{\frac{d}{d\lambda}}\left\{\frac{\mathrm{az}}{\left(\mathrm{ax}+\mathrm{ay}\right)^{\prime}}\right\}\{{\mathrm{Cz}\}}\right]/\mathrm{P}^{\prime}_{\left({\lambda}\mathrm{CP}\right)}]*\mathrm{P}_{\left({\lambda}\mathrm{CP}\right)}$$

Thus, {Z} concentrations in the mixtures are calculated utilizing the regression equation demonstrating the correlation among the amplitude of the recovered derivative ratio spectra using normalized spectra of (X + Y) at graphical Peak to Zero (P - zero) or peak to peak measurement(P_max−min_)and the correlating concentration of pure Z.

The concentrations of X and Y can also be obtained by applying the same procedure steps using the corresponding double divisors (Y + Z) in case of X and (X + Z ) in case of Y.

#### Automated double divisor derivative ratio (AUTO-DD-DD)

The AI-assisted approach simplifies and speeds up spectrophotometric data handling by reducing manual steps and minimizing errors. Instead of relying on time-consuming, manual operations in Spectra Manager^®^ software, the complete data is fed directly into an AI model via a, CSV file. With expert guidance, the AI instantly performs the necessary analyses and transformations, ensuring quick, accurate results. This method enhances efficiency and reduces the likelihood of errors compared to traditional workflows.Allprompts and decisions based on the theoritical background of the applied method were monitored by expertise analyst,

**Steps for AI-assisted data handling Using ChatGPT**

- Analysis of Z in X + Y+Z combination.
- Solvent: Methanol.
- Instrument: UV-Vis Spectrophotometer.
- Software: ChatGPT, an AI-powered tool, allscanned spectra are input in Chat GPT as, CSV files (Microsoft Excel Comma Separated Values File).

- **Step 1: Construct regression equation**: Input the data of scanned spectra of different concentrations of pure Z (Column A) and equimolar of (X + Y) (Column B).

- Use a prompt:

> *“Use the ratio Column A versus column B then get derivativeusing Δ λ = 4 and scaling factor 10thenselect optimum wavelength without any contribution of column B*,* generateregression equation at this wavelength and calculate the recovery % of Z and SD%*,* LOD and LOQ.”*

- **Step 2: Confirm accuracy**: Input the data of scanned spectra of threedifferent concentrations of pure Z.

- Use a prompt:

> *“Use the generated regression equation and calculate the recovery % of Z using AI-generated regression equation at specifiedwavelength and calculate SD.*

- **Step 3: Confirm precision**: Input the data of scanned spectra of threedifferent concentrations of pure Z in two different columns (Intraday and Interday).

- Use a prompt:

> *“Use the derivative ratio spectra of each pure Z column D and F the spectral data of column B in division and calculate the recovery % of Z using AI- generated regression equation at specified wavelength and calculate RSD”.*

- **Step 4: Confirm specificity** Input the data of scanned spectra of different synthetic mixtures of X, Y and Z.

- Use a prompt:

>*“Apply derivative of ratio spectra of mixture using the spectral data of column Bin divisionusing Δ λ = 4 and scaling factor 10.”*

- **Step 5: Analysis of pharmaceutical formulation**: Input the data of scanned spectra of extracted pharmaceutical formulation.

- Use a prompt:

>*“Apply derivative of ratio spectra of samples using the spectral data of column B in division.using Δ λ = 4 and scaling factor 10”*.

## Materials and methods

### Instrumentation and softwares

Spectral measurements are done using a double-beam UV/Visible spectrophotometer; V-760, Jasco, Japan, and ACER compatible computer with software (Microsoft excel 2010). Scans are done in a 1.00 cm quartz cells in the range from 200 to 400 nm at room temperature. Softwares, Spectra Manager^®^ software, JASCO corporation, version 2. ChatGPT (OpenAI; web-based interface, GPT-5.1 architecture, accessed between [July–August 2025])andMicrosoft Copilot (web-based interface, powered by GPT-4, accessed between [July–August 2025])which are a free of charge AI version.

### Chemicals and reagents

#### Pure samples

CLIO and TOL were obtained from Alexandria Company for Pharmaceuticals, with measured purities of 99.96 ± 1.04 and 100.07 ± 0.60, respectively, as confirmed by the British Pharmacopoeia (BP) official procedures^17^, BETA and CC were supplied by Memphis Company, showing purities of 99.65 ± 1.16 and 100.09 ± 1.09, respectively, also determined in accordance with BP methods^17^, GEN was provided by Sigma Pharmaceuticals, with a purity of 100.70 ± 0.78, verified using the United States Pharmacopeia (USP) official method^18^ .

#### Pharmaceutical formulation

Quadriderm cream^®^ (Memphis Company for Pharmaceuticals, Egypt) was obtained from the local market. Each gram of the formulation is labeled to contain 10.0 mg of CLIO, 10.0 mg of TOL, 0.5 mg of BETA, 1.0 mg of CC, and 1.0 mg of GEN.

#### Standard solution

Stock standard solutions were prepared at a concentration of 250.0 µg/mL for (CLIO, TOL, BETA, and CC) in methanol and for (GEN) in bidistilled water. From these stocks, working solutions were created at lower concentrations: BETA and CC at 50.0 µg/mL in methanol, CLIO and TOL at 25.0 µg/mL in methanol, and GEN at 25.0 µg/mL in bidistilled water.

### Procedure

#### Preparation of spectral data

- Zero-order spectra of CLIO, TOL, BETA, and CC (5.0 µg/mL) were scanned from 200 to 400 nm using methanol as a blank.
- The factorized TOL spectrum was obtained by division of the DD^1^(TOL/(CC+BETA)’] by the value of thepeak amplitude at 222 nm using Δ λ = 4 and scaling factor 10and saved on the PC.
- The factorized CC spectrum was obtained by division of the DD^1^[CC/(BETA + TOL)’] by the value of the peak amplitude at 242.6 nm using Δ λ = 4 and scaling factor 10and saved on the PC.

#### Linearity and calibration graphs

Aliquots corresponding to 0.5–5.0 µg/mL of TOL, 3.0–30.0 µg/mL of BETA, and 2.0–20.0 µg/mL of CC were taken from their respective working solutions (25.0 µg/mL for TOL, 50.0 µg/mL for BETA, and CC), transferred into separate 10-mL volumetric flasks, and diluted with methanol. The standards were then scanned from 200 to 400 nm against methanol as a blank, with spectra recorded digitally.

- **For TOL**: D^0^spectra for TOL are divided by normalized spectrum of (BETA + CC)’as double divisor, and then the first derivative is obtained. The calibration graph is made by relating the P_max_ at 225.2 nm in case of **MAN-DD-DDE** and coincidence point of at 222.0 nm in case **MAN-DD-DD** versus the corresponding concentration.
- **For CC**: D^0^spectra for CC are divided by normalized spectrum of (BETA + TOL)’as double divisor and then the first derivative is obtained. The calibration graph is made by relating the P_max-min_ at 236.6 -227.0nmin case of **MAN-DD-DDE** and coincidence point of at 242.6 nm in case **MAN-DD-DD** versus the corresponding concentration.
- **For BETA by MAN-DD-DD**: D^0^spectra for BETA are divided by normalized spectra of (TOL + CC)’ as double divisorand then the first derivative is obtained. The calibration graph is made by relating the P_max_ at 250.0 nm versus the corresponding concentration.
- **BETA by AUTO-DD-DD**: D^0^spectra for BETA and normalized spectra (TOL + CC)’as double divisor are uploaded to ChatGPTas, CSV files. The AI will compute the first derivative of the ratio spectra for each concentration, then plot the peak amplitude at 250.0 nm against concentration to create a calibration curve with its regression equation.

#### Application of spectrophotometric methods for CLIO, TOL, BETA, and CC in synthetic mixtures

- Synthetic mixtures with different ratios of CLIO, TOL, BETA, and CC were analyzed using Spectra Manager^®^by dividing their spectra by the normalized D^0^ spectrum of CLIO. The CLIO concentration was determined using a constant obtained in the 340.0–355.0 nm region, multiplied by the normalized spectrum via the CM method, and calculated from the regression equation at 254.8 nm. The CLIO spectrum was then subtracted from the mixture to obtain a ternary subsystem of BETA, TOL, and CC.

**For Manual Strategy via Spectra Manager**^®^.

- **For TOL**: The resolved ternary mixture is divided by normalized spectrumof(BETA + CC)’ as double divisor, then the first derivative was obtained. The amplitude at 222.0 nm is either substituted in the corresponding equation or was multiplied by the factorized TOL spectrum, and the amplitude at P_max_ 225.2 nm was substituted in the corresponding regression equationto get the concentration of TOL in the mixture.
- **For CC**: The resolved ternary mixture is divided by normalized spectrumof(BETA + TOL)’ as double divisor, then the first derivative was obtained. The amplitude at 242.6 nm is either substituted in the corresponding equation or was multiplied by the factorized CC spectrum, and the amplitude at P_max_236.6 nm was substituted in the corresponding regression equationto get the concentration of CC in the mixture.

##### For BETA

The resolved ternary mixture is divided by normalized spectrumof (TOL + CC)’as double divisor, then the first derivative was obtained. The amplitude at 250.0 nm is substituted in the corresponding equation to obtain BETA concentration.

- **For AUTO Strategy via ChatGPT**.
- Zero-order absorption spectra of the resolved ternary mixtures of (BETA + TOL+CC) are uploaded to **ChatGPTas**,** CSV files**, in addition to the normalized double divisor spectrum (TOL + CC)’, csv file. The AI is asked to get the first derivative of the ratio spectrum of each concentration, measuredpeak amplitude at P_min_ 250.0 nm is and substituted in the regression equation to get the concentration of BETA in the mixture.

#### Application to pharmaceutical formulation

An accurately weighed 2.0 g portion of the cream was placed in a clean, dry beaker, spiked with 12.0 mg BETA and 8.0 mg CC, and dissolved in 20.0 mL methanol by heating on a 70 °C water bath for 5 min until melted. The mixture was transferred into three screw-capped test tubes, and the beaker was rinsed with an additional 10.0 mL of methanol. The tubes were vigorously shaken using a vortex mixer until resolidification occurred, then reheated at 70 °C for 5 min to remelt the sample. This melt–shake cycle was repeated three times to ensure complete extraction of all four components. The resulting solution was frozen for 20 min, centrifuged for 5 min, and filtered cold into a calibrated measuring cylinder. The filter paper was rinsed with 10.0 mL methanol, affording a total of 40.0 mL stock solution containing CLIO and TOL (500.0 µg/mL each), BETA (325.0 µg/mL), and CC (250.0 µg/mL). From this stock, 0.1 mL was accurately transferred into a 10.0-mL volumetric flask and diluted to volume with methanol, yielding a working solution containing 5.0 µg/mL CLIO and TOL, 3.25 µg/mL BETA, and 2.5 µg/mL CC. This solution was then subjected to the same analytical procedures described for laboratory-prepared mixtures.

## Results and discussion

**Fig. 1:**
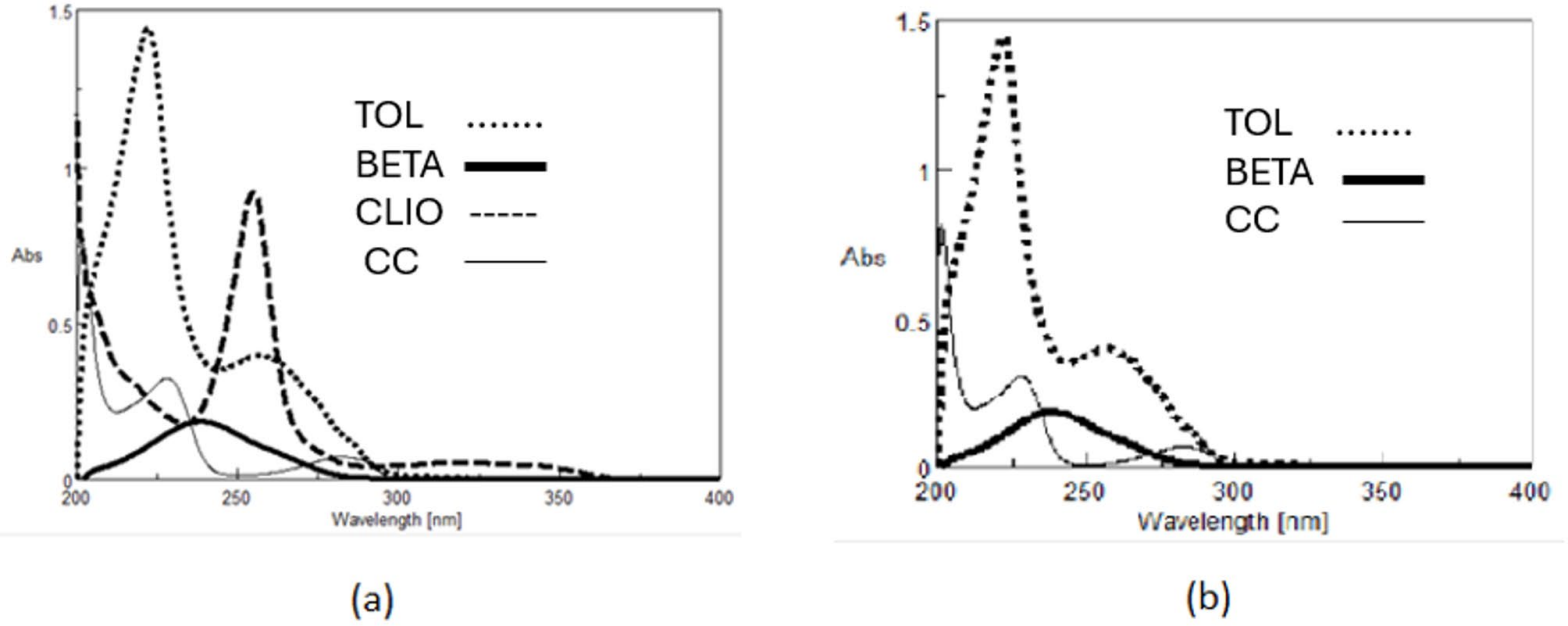
(**a**) Zero order absorption spectrum of TOL, BETA, CLIO, and CC; 5.0 µg/mL each dissolved in methanol. (**b**) Zero order absorption spectrum of TOL, BETA, CC; 5.0 µg/mL each dissolved in methanol.

UV–Vis spectrophotometry was previously used to analyze a quaternary mixture, successfully separating CLIO and GEN, with GEN determined via liquid extraction and fluorimetry, and a methanolic extract containing CLIO, TOL, BETA, and CC analyzed through spectrum subtraction to resolve the ternary sub-mixture of BETA, CC, and TOL^1^,^19^. (Figure 1a and b). The earlier protocols, including calibration and sample preparation details, are documented in the Supplementary File. In the current study, the focus shifted to a more challenging ternary mixture of TOL, BETA, and CC, for which a new analytical method was developed. The workflow involved applying previous protocols for CLIO and GEN, implementing the new method for TOL, BETA, and CC, and validating the data through calibration, linearity, and performance assessments.

Zero-order absorption spectra of the quaternary mixture of CLIO, BETA, TOL, and CC. The first component CLIO shows an extended spectrum in the region 340.0–355.0 nm, where the other three components show zero contribution as shown Fig. 1. The whole mixture shows severe overlap from 200.0 to 290.0 nm. Depending on the presence of that extended region where only CLIO has a contribution, constant multiplication coupled with spectrum subtraction (CM-SS)^20^ was applied to resolve the ternary mixture of BETA, TOL, and CC.

Each component in the ternary mixture (TOL + BETA + CC) is resolved alone by the double divisor derivative ratio method (MAN-DD-DD) using equimolar double divisors of the two components we need to eliminate.The methods used are summarized in scheme (1).

**Figure:**
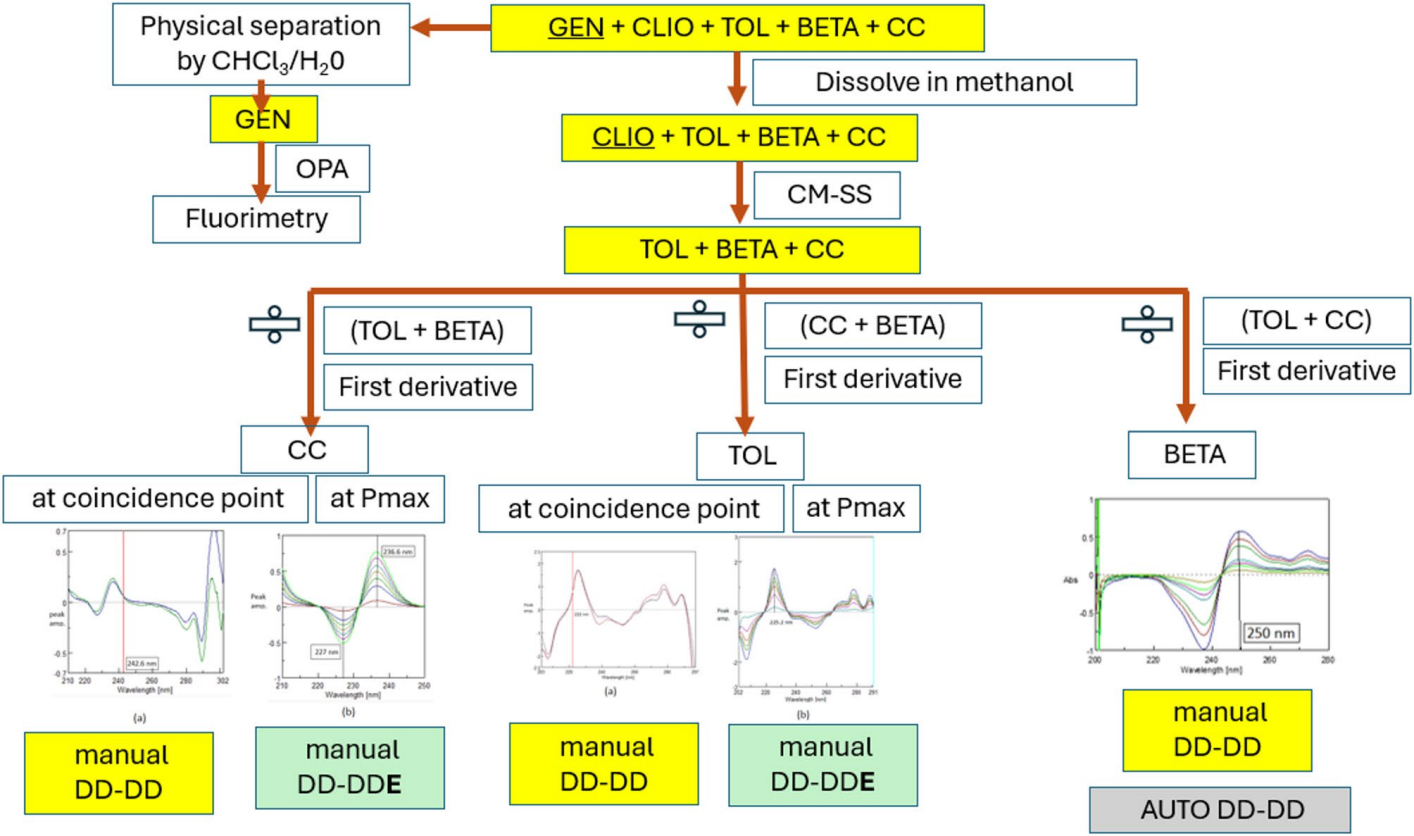
Scheme 1.

**Manual double divisor derivative ratio [MAN-DD-DD]**

**Fig. 2:**
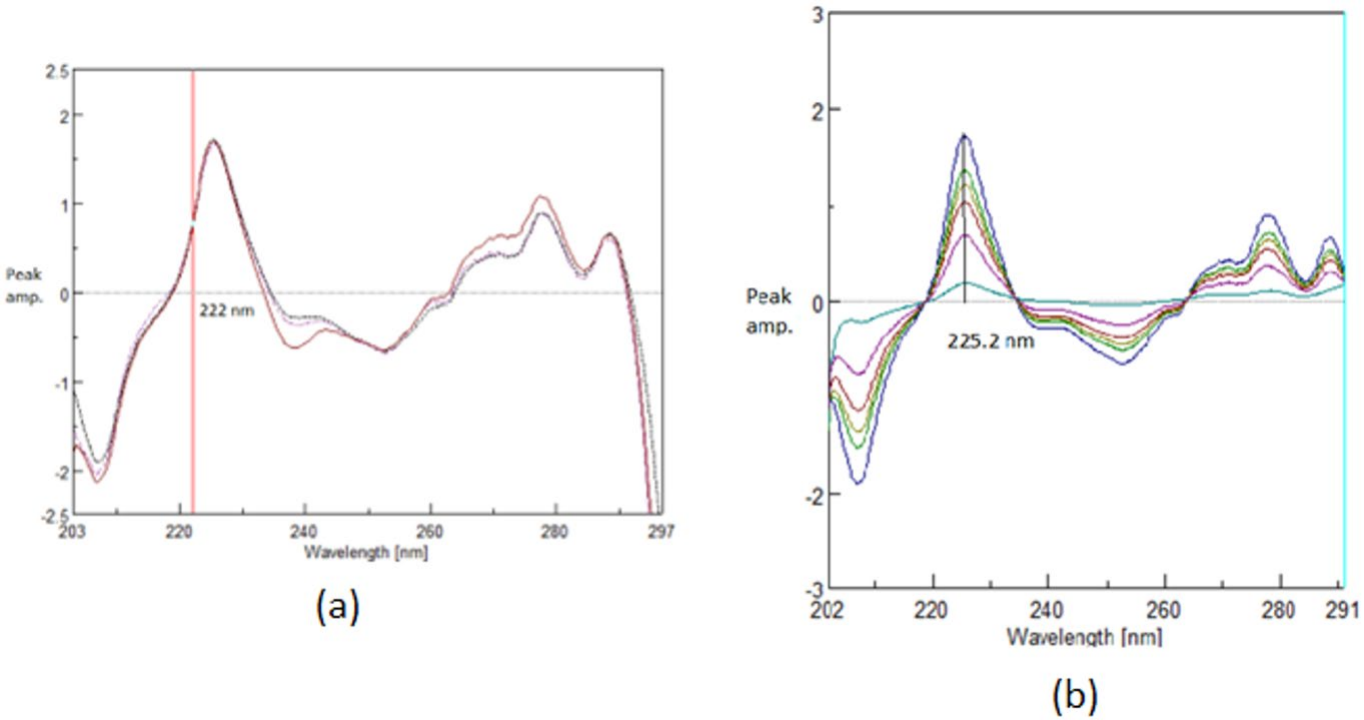
First derivative of the ratio spectrum using normalized spectrum of (BETA + CC)’ as double divisor for (**a**) Ternary mixture (TOL 5.0 µg/mL, BETA 15.0 µg/mL, CC 10.0 µg/mL), (TOL 5.0 µg/mL, BETA 5.0 µg/mL, CC 5.0 µg/mL) and pure TOL (5.0 µg/mL) showing a coincidence point at 222.0 (**b**) Concentrations of pure TOL (0.5–5.0 µg/mL) at 222.0 via Spectra Manager^®^ in case of DD-DDE method.

**Fig. 3:**
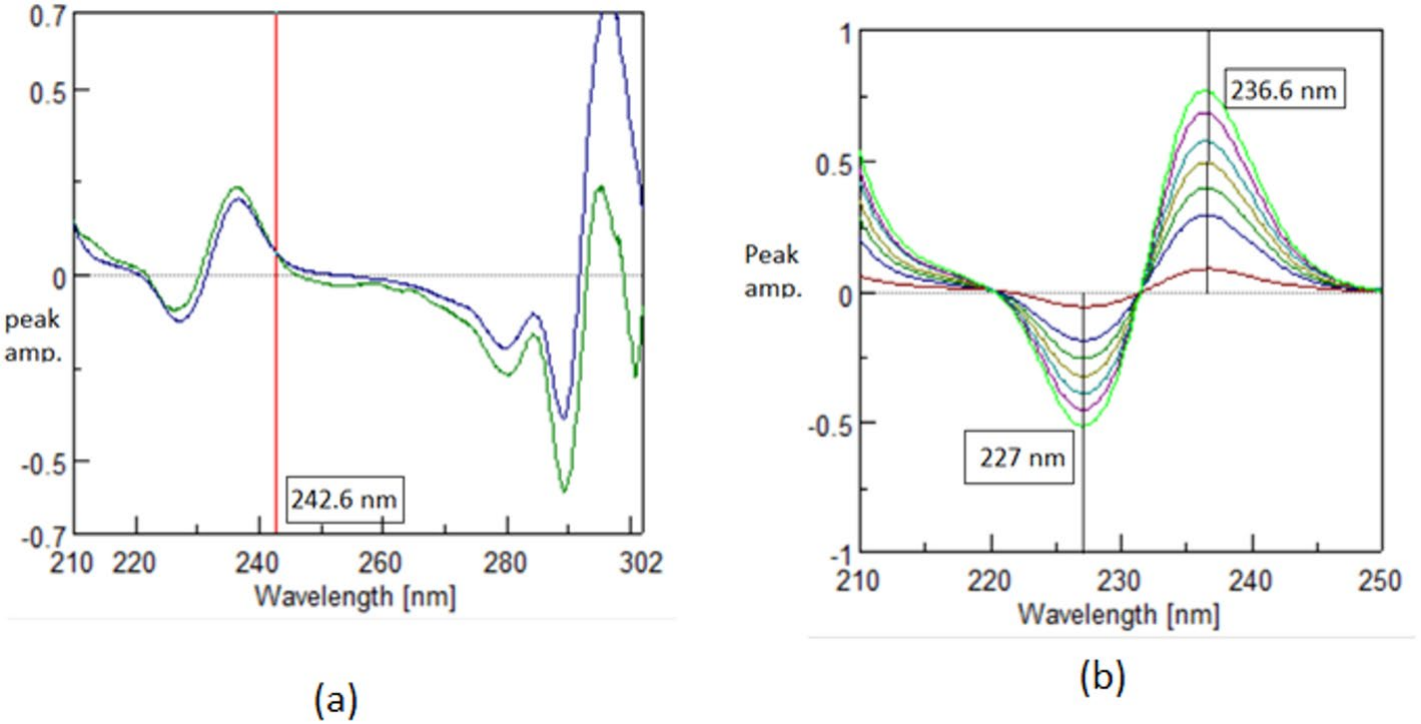
First derivative of the ratio spectrum using normalized spectrum of [BETA + TOL]’ as double divisorfor (**a**) Ternary mixture (TOL 5.0 µg/mL, BETA 5.0 µg/mL, CC 5.0 µg/mL) and pure CC(5.0 µg/mL) showing a coincidence point at 242.6 nm (**b**) Concentrations of pure CC(2.0–20.0 µg/mL) at 242.6 nm via Spectra Manager^®^ in case of DD-DDE method.

The double divisor derivative ratio spectrophotometric (DD-DD) method, utilizing Spectra manager^®^ software, effectively analyzes ternary mixtures by reducing spectral overlap and improving accuracy. It is especially useful when component spectra overlap significantly. For TOL and CC, this method identifies a coincidence point at the spectrum shoulder, enhancing reliable quantification. Figures (2 and 3).

**Fig. 4:**
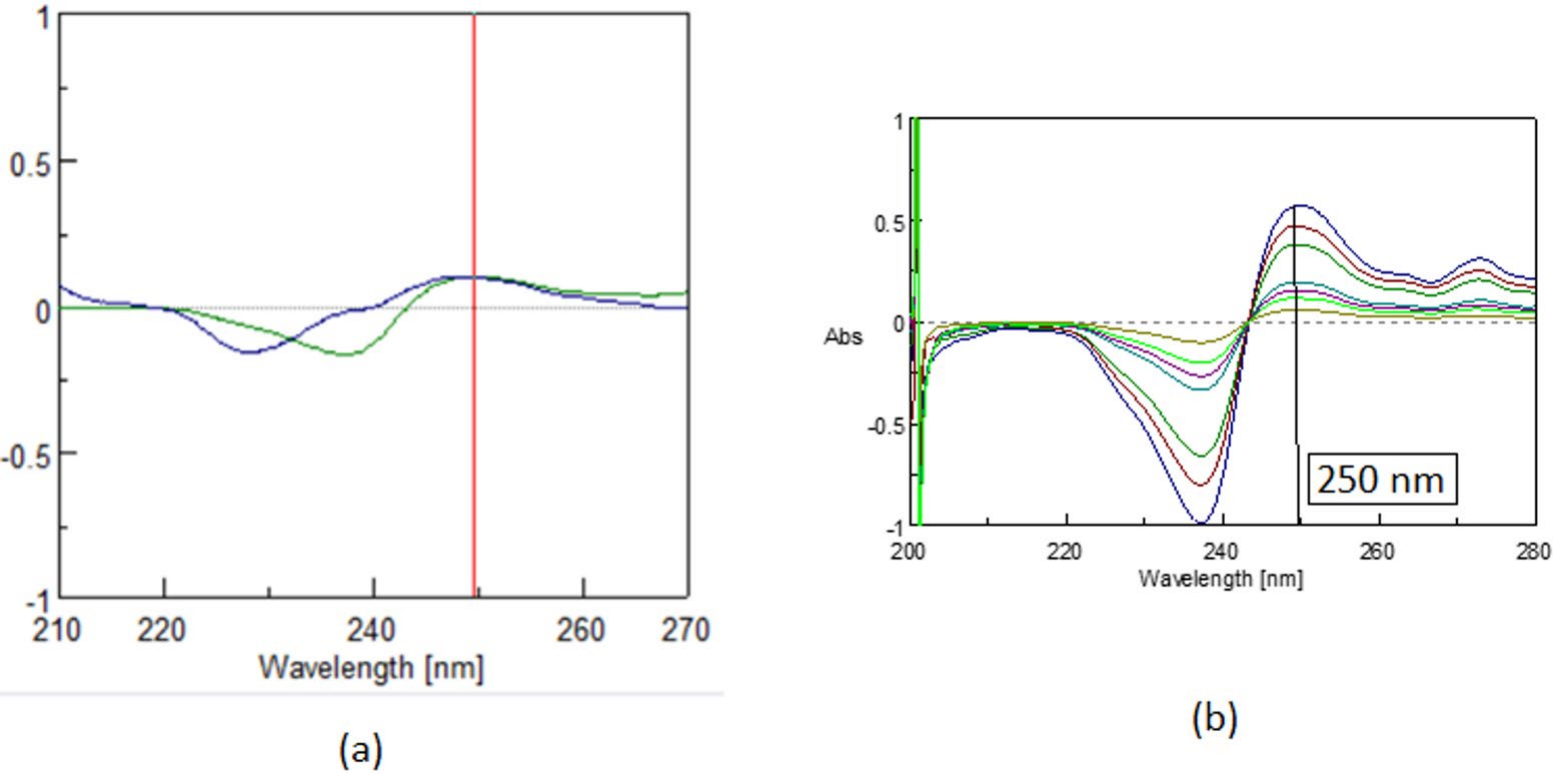
First derivative of the ratio spectra using normalized spectrum of [CC + TOL]’ as double divisor for: (**a**) Ternary mixture (TOL 5.0 µg/mL, BETA 5.0 µg/mL, CC 5.0 µg/mL) and pure BETA (5.0 µg/mL) showing a coincidence point at 250.0 nm. (**b**) Concentrations of pure BETA (3.0–30.0 µg/mL) in case of DD-DDE method.

The traditional derivative ratio double divisor method^16^ is applied for the determination of TOL, CC and BETA using a double divisor of normalized spectrum of [CC + BETA]’, [BETA + TOL]’ and [CC + TOL]’ the coincidence point used in the construction of the calibration curve is found to be 222.0 nm, 242.6 nm and 250.0 nm, respectively as shown in Figs. 2a, 3a and 4a and the results are shown in Table 1.

The critical measurement of amplitudes of in case TOL and CC at 222.0 nm and 242.6 nm is restricted to the shoulder region, rather than the peak maximum (P_max_ or P_min_). To account for this limitation, the DD-DDE method must be employed, which involves coupling with a factorized spectrum to enhance the robustness of the measurement. While in case of BETA; the coincidence point represents P_min_ at 250.0 nm as shown in Fig. 4; so the conventional DD-DD is applied with good sensitivity and robustness.

**Table 1:** Assay parameters and method validation obtained by applying the proposed spectrophotometric methods.

Parameter	TOL		BETA		CC		CLIO	GEN
	[MAN-DD-DDE] (*P*_225.2 nm_)	MAN-DD-DD (*P*_222 nm_)	MAN-DD-DD (*P*_250.0 nm_)	AUTO-DD-DD (*P*_250.0 nm_)	[MAN-DD-DDE] (*P*_236.6–227 nm_)	MAN-DD-DD (*P*_242.6 nm_)	D^0^ (254.8 nm)	Fluorimetry λ_em_ 419 nm
Range(µg/mL)	0.5-5.0		3.0–30.0		2.0–20.0		1.0–7.0	0.25–1.25
Slope	0.3406	0.1575	0.019	0.0207	0.0637	0.0111	0.1529	72.076
Intercept	-0.0072	-0.0182	0.0015	-0.0123	0.0134	0.0033	-0.0113	0.1954
Correlation coefficient (r)	0.9997	0.9997	0.9999	0.9996	0.9999	0.9999	0.9999	0.9999
Accuracy Mean ± SD	100.62 ± 1.80	97.89 ± 1.45	99.85 ± 0.99	98.00 ± 0.01	99.59 ± 1.87	99.15 ± 1.40	100.08 ± 1.67	100.22 ± 0.32
Precision								
Intraday(RSD^a^)	0.835	0.901	0.545	0.016	1.005	1.208	0.563	0.429
Interday(RSD^b^)	1.461	4.991	1.467	0.046	1.817	1.303	0.780	1.858
LOD(µg/mL)	0.09	0.09	0.26	0.30	0.21	0.36	0.07	0.01
LOQ (µg/mL)	0.28	0.28	0.80	0.90	0.65	1.10	0.22	0.04

RSD^a^&RSD^b^: both intra-day& inter-day (*n* = 9) three concentrations in triplicate. Relative standard deviations were held of concentrations 3.0, 4.0, 5.0 µg/mL for TOL, 7.5, 12.5, 17.5 µg/mL for CC, 5.0, 10.0, 15.0 µg/mL for BETA, 3.0, 4.0, 5.0 µg/mL CLIO, and 0.25, 0.375, 1.00 µg/mL for GEN.

**MAN-DD-DDE**

The idea of Manual Double Divisor Derivative Ratio Extraction emerged to get the whole derivative ratio spectrum of either TOL or CC, allowing its determination at maxima or peak-to-peak P_max-min_.

In case of TOL to obtain (TOL/[CC+BETA]’ ) alone, allowing construction of the calibration curve at P_max_ 225.2 as shown in Fig. 2(b) while in case of CC to obtain (CC/[TOL+BETA]’ ) alone, allowing construction of the calibration curve at peak to peak P_236.6–227.0 nm_ as shown in Fig. 3(b).

**AUTO-DD-DD**

Our main goal in automation using AI tools is to streamline the MAN-DD-DD process by accelerating and simplifying its manipulation steps. To achieve this, we are introducing free and user-friendly AI solutions into method development and optimization. However, to ensure the accuracy and effectiveness of AI tools, expert analysts must actively collaborate with the AI system, providing carefully crafted prompts and closely monitoring each generated output. This interactive process requires analysts to request specific evidence and feedback at every stage, enabling them to make informed decisions and guarantee the reliability of the AI-driven spectrophotometric method development process. The AUTO steps as follows :

- Software: ChatGPT, an AI-powered tool, All scanned spectra are input in ChatGPTas, CSV files (Microsoft Excel Comma Separated Values File.

- **Step 1: Construct regression equation**: Input the data of scanned spectra of 3.0–30.0 of pure BETA in region 200–300 nm (Column A) and normalized spectrum of (TOL + CC) (Column B).

- Use a prompt:

*> “Use the ratio Column A versus column B then get derivative then select optimum wavelength without any contribution of column B*,* generate regression equation at this wavelength and calculate the recovery % of Z and SD%*,* LOD and LOQ.”*

***Output: Results were mentioned in*** Table 1***using 250 nm as optimum wavelength*** (Fig. 6).

- **Step 2: Confirm accuracy**: Input the data of scanned spectra of three different concentrations of pure Z.

- Use a prompt:

*> “Use the generated regression equation and calculate the recovery % of Z using AI- generated regression equation at specified wavelength and calculate SD.*

***Output: Results were mentioned in*** Table 1.

- **Step 3: Confirm precision**: Input the data of scanned spectra of threedifferent concentrations of pure Z in two different columns (Intraday and Interday).

- Use a prompt:

*> “Use the derivative ratio spectra of each pure Z column D and F the spectral data of column B in division and calculate the recovery % of Z using AI- generated regression equation at specified wavelength and calculate RSD .”*

***Output: Results were mentioned in*** Table 1.

- **Step 4: Confirm specificity**: Input the data of scanned spectra of different lab mixtures of X, Y and Z.

- Use a prompt:

>*“Apply derivative of ratio spectra of mixture using the spectral data of column B in division”*.

***Output: Results were mentioned in*** Table 2.

- **Step 5: Analysis of pharmaceutical formulation**: Input the data of scanned spectra of extracted pharmaceutical formulation and those of standard addition.

- Use a prompt:

>*“Apply derivative of ratio spectra of samples using the spectral data of column B in division*.

***Output: Results were mentioned in*** Table 2.

**Fig. 5:**
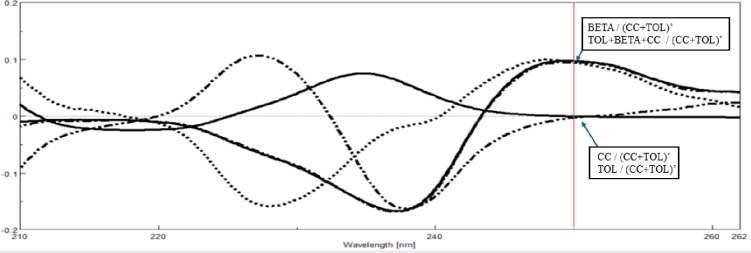
First derivative of the ratio spectrum using normalized spectrum of (CC + TOL)’ as double divisor of ternary mixture 5.0 µg/mL each (TOL + BETA + CC), pure BETA (5.0 µg/mL) and both CC and TOL showing a coincidence point at 250.0 nm via Spectra Manager^®^.

**Fig. 6:**
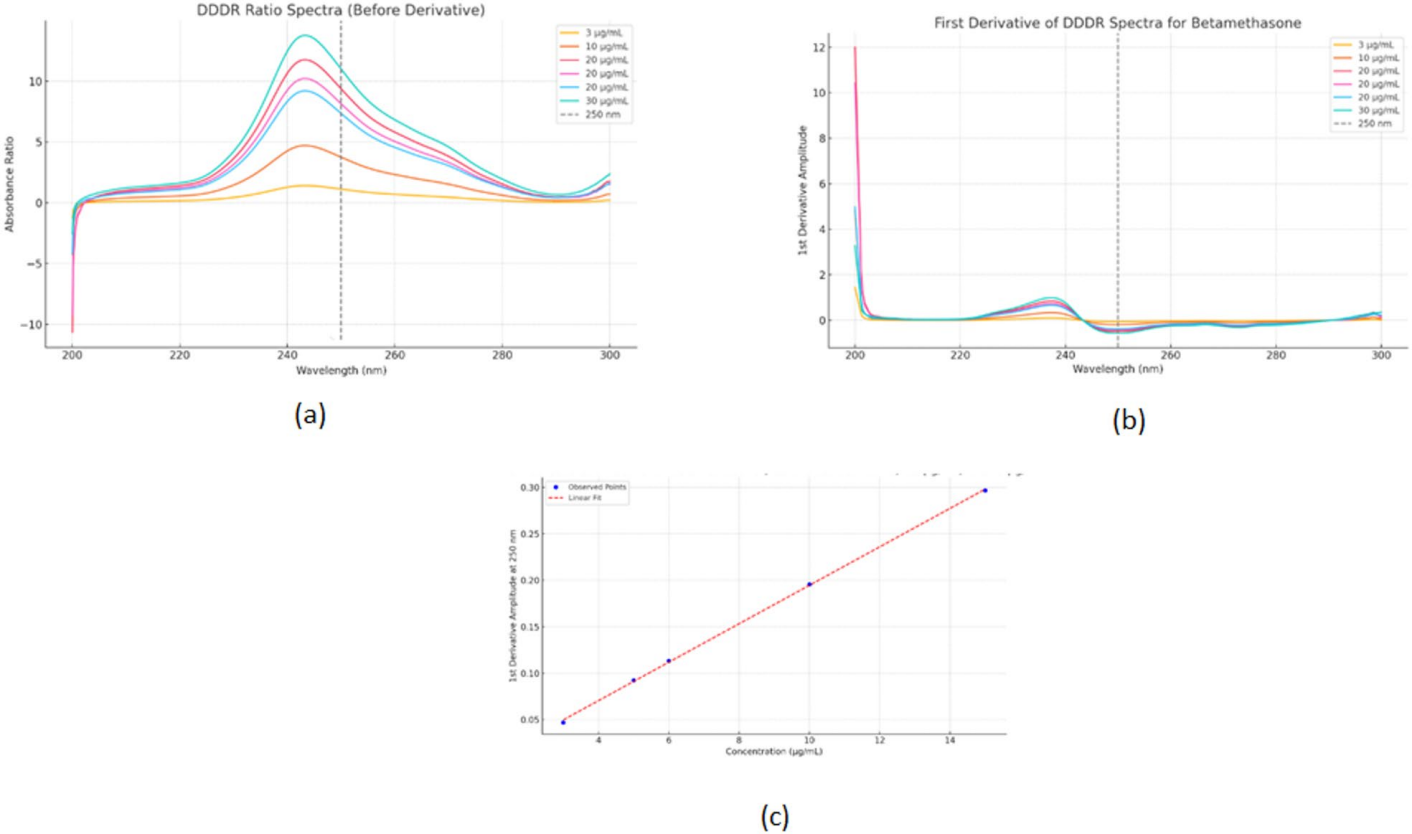
AI generated figures of ratio spectra (**a**), their first derivative (**b**) and obtained calibration graph at 250.0 nm versus concentrations (**c**) of several BETA concentrations using normalized spectrum of (TOL + CC)’.

We uploaded different concentrations of pure BETA, TOL, and CC as, CSV(input ), in addition to the normalized spectrum of [CC + TOL]’ as double divisor. Chat GPT processed the files by four successive prompts, First one to divide BEA, TOL and CC on [CC + TOL]’, second one to get the first derivative of each output ratio spectra, then ask to detect the wavelength where TOL and CC have no contribution (It chose 250 nm as the coincidence point which comply with that obtained by Spectra Manager^®^ software based on the theoretical background of the suggested method where BETA has a value while TOL and CC showed zero region at this wavelength as shown in Fig. 5 and finally plot a calibration curveat250.0 nm, and apply validation on the uploaded scanned spectra for accuracy and precision. ChatGPT also ranked the best wavelengths to be used in the construction of the calibration curve, ranked by linearity r^2^ as shown in the Table S1. The method was explained in text, but the results were not satisfying at first, not matching the results obtained from the Spectra Manager^®^ software, so screenshots of the steps performed on Spectra Manager^®^ were uploaded to ChatGPT to make it learn the steps and optimize its method. As the results were still not satisfactory, we asked to just work on the overlapped region 200.0–300.0 nm, and also uploaded five mixtures declaring their BETA concentrations. The AI model generated the calibration curve, eliminated the outlier concentrations, and deduced the regression equation. The AI model also could get the MAN-DD-DD linearity figures of BETA in the range 3.0–30.0 µg/mL showing a P_min_ at 250.0 nm where the calibration graph was constructed as shown in Fig. 6. The same amplitudes values of the derivative spectra were obtained by SpectraManager^®^ and those generated by AI at 250.0 nm, with the only difference being their direction, confirming that there was no interference from noise.

ChatGPT was used to perform calculations on BETA, CSV spectra. It was instructed to perform DD-DD analysis, substitute amplitude at 250.0 nm Fig. 6(c), and calculate R% for each concentration. Results were obtained within minutes, matching accepted values from manual calculations in Microsoft Excel and Spectra Manager software, with significant time savings.

Focusing initially on BETA helps maintain clarity and ensures thorough validation, which prevents potential issues with the manuscript’s tone. Once the method is developed and validated for one component, it can be efficiently extended to others. The application of the AUTO DD-DD method to BETA is not a narrow extension; it represents a central part of a broader innovation that includes automated divisor selection and rapid spectral processing.

The results obtained from the novel DD-DDE, MAN-DD-DD and AUTO-DD-DD were compared in Table 2.

**Table 2:** Determination of BETA, CC, TOL, CLIO, and GEN in laboratory prepared mixtures and pharmaceutical dosage form.

Conc. (µg/mL)	TOL		BETA		CC		CLIO	GEN
	[MAN-DD- DDE] (*P*_225.2 nm_)	MAN-DD-DD (*P*_222 nm_)	MAN-DD-DD (*P*_250.0 nm_)	AUTO-DD-DD (*P*_250.0 nm_)	[MAN-DD- DDE] (*P*_236.6–227 m_)	MAN-DD-DD (*P*_242.6 nm_)	D^0^ (254.8 nm)	Fluorimetry λ_em_ 419 nm
Synthetic mixtures	Recovery % ± SD							
Mixture1	100.02± 0.03	100.40 ± 0.03	97.95±2.16	100.94±0.31	99.21± 2.94	99.15 ± 1.59	100.36 ± 1.09	98.87± 0.41
Mixture 2	97.87±0.67	98.28±0.67	100.57±0.56	98.79±0.88	103.40±1.75	101.91± 1.03	99.87±0.41	100.61± 0.35
Mixture 3	97.78±0.30	98.17 ± 0.29	100.68±1.97	103.84±1.47	96.92± 0.09	97.81 ± 0.47	100.86 ± 0.48	100.20±0.68
Mixture 4	100.59± 0.05	102.85 ± 0.05	97.69 ± 1.31	102.23±0.98	100.39±0.20	100.99± 0.31	101.52 ± 0.50	100.89±0.70
DF ratio^a^ mixture	100.93±0.18	101.30 ± 0.18	96.54± 0.02	101.23± 0.87	96.88±0.58	101.33± 1.94	98.97± 0.66	99.49± 0.66
Dosage form Found% ± SD	104.78± 1.27	104.72 ± 1.17	98.37 ± 0.54	98.82±0.98	96.94±0.66	98.17±1.07	99.69± 0.35	99.68± 0.66

^a^Ratio present in the dosage form and (Quadriderm^®^) Batch no.: ET-15-YNCZ-124 5.0 : 0.25^b^ :0.5 ^c^:5.00 :0.5 after sample enrichment using 3.00 µg/mL and 2.00 µg/mL of pure BETA and CC, respectively.

^b^After subtraction of the added BETA.

^c^After subtraction of the added CC.

AUTO results is the average of three different prompts.

**Validation**

The proposed methods either manual or automated were validated according to ICH guidelines^21^. The integration of Artificial Intelligence (AI) into analytical workflows complements traditional method validation, but does not replace it. The integration of Artificial Intelligence (AI) into analytical workflows has significantly streamlined data manipulation, allowing for faster and more consistent results. Despite this efficiency gain, all methods remain subject to stringent validation protocols, including assessments of accuracy, precision, and robustness. To ensure the highest standards of quality and reliability, experienced analytical chemists closely supervise and guide all AI-assisted steps. This approach not only reduces analysis time but also maintains compliance with regulatory requirements, making it a highly suitable solution for routine quality control laboratories. Analytical calibration curves were found to be linear for the three compounds, demonstrating suitability for routine use. Accuracy was confirmed with high recovery rates for all three compounds, and precision studies showed good reproducibility (Tables 1 and 2).

## Statistical analysis

The proposed spectrophotometric methods were statistically compared to the BP official procedures using Student’s t-test, F-test, and ANOVA. The results confirmed that there were no significant differences in accuracy or precision between the two approaches. The findings were presented in Tables 3 and 4, and were also supported by ANOVA analysis against literature values. The analysis showed no significant difference between the proposed methods and those reported in the literature^1^ this confirms the reliability and accuracy of the proposed methods. The E% was also calculated showing very low error. These findings are presented in Tables 5 and 6.

**Table 3:** Statistical comparison between results obtained by the proposed methods and the official methods for determination of TOL, BETA, CC, CLIO, and GEN in pure powder form.

Drug	Method	Mean (%)	S.D.	*n*	Variance	F-test (5.05)	Student’s t-test (2.23)
TOL	MAN-DD-DDE P_225.2 nm_	100.27	1.33	6	1.78	1.13	0.23
	MAN-DD-DD at P_222nm_	100.38	1.63	6	2.68	1.69	0.33
	Official method^a^	100.10	1.26	6	1.59	–	–
BETA	MAN-DD-DD at P_250 nm_	98.30	1.24	6	1.54	1.12	1.17
	AUTO-DD-DD at P_250 nm_	99.21	1.16	6	1.35	1.01	1.30
	Official method^a^	99.21	1.17	6	1.37	–	–
CC	MAN-DD-DDEP_236.6–227 nm_	100.13	1.28	6	1.63	2.76	0.01
	MAN-DD-DD p_242.6_ nm	99.83	1.43	6	2.04	3.46	0.42
	Official method^b^	99.88	0.77	6	0.59	–	–
CLIO	Official method^c^	99.78	1.35	6	1.83	1.68	0.25
GEN	Official method^b^	99.96	1.04	6	1.09	–	–

^a^Spectrophotometric method for the determination of TOL at 257 nm and BETA at 238.5 nm.

^b^HPLC method.

^c^Titrimetric methods.

**Table 4:** One way ANOVA testing for the proposed methods and the USP official methods used for the determination of TOL, BETA, and CC in pure powder form.

	Source of variation	Sum of squares	Degree of freedom	Mean square	F	F critical
TOL	Between Groups	0.24	2.00	0.12	0.06	3.68
	Within Groups	30.24	15.00	2.01		
	Total	30.49	17.00			
BETA	Between Groups	7.55	2.00	3.77	3.24	
	Within Groups	17.48	15.00	1.17		
	Total	25.03	17.00			
CC	Between Groups	0.37	2.00	0.19	0.11	
	Within Groups	24.63	15.00	1.64		
	Total	25.00	17.00			
CLIO	Between Groups	2.48	1.00	2.48	0.22	4.96
	Within Groups	14.54	10.00	1.45		
	Total	17.02	11.00			
GEN	Between Groups	1.38	1.00	1.38	3.00	
	Within Groups	4.59	10.00	0.46		
	Total	5.97	11.00			

**Table 5:** Statistical comparison between results obtained by the proposed methods and the reported method^1^ for determination of TOL, BETA, and CC in quadriderm cream.

Conc. (µg/mL)	TOL			BETA			CC		
	[MAN-DD-DDE] (*P*_225.2 nm_)	MAN-DD-DD (*P*_22 nm_)	Reported method [1]	MAN-DD-DD (*P*_250.0 nm_)	AUTO-DD-DD (*P*_250.0 nm_)	Reported method [1]	[MAN-DD- DDE] (*P*_236.6–227 m_)	MAN-DD-DD (*P*_242.6 nm_)	Reported method [1]
Mean ± SD	104.78 ± 1.27	104.72 ± 1.17	102.73 ± 1.07	98.37 ± 0.54	98.82 ± 0.98	99.86 ± 0.40	96.94 ± 0.66	98.17 ± 1.07	98.07 ± 0.42
variance	1.62	0.70	1.16	0.43	1.15	0.17	0.30	0.96	0.16
n	3	3	3	3	3	3	3	3	3
Student-t test (2.77)	2.12	2.43	–	0.06	1.20	–	2.51	0.16	–
F-test (19)	1.39	1.66	–	1.80	2.00	–	1.01	6.61	–
E% (Found conc -claimed conc)/claimed conc	0.047	0.046	–	0.016	0.011	–	0.030	0.018	–

**Table 6:** One way ANOVA testing for the proposed methods and the reported method^1^ used for the determination of TOL, BTA, and CC in quadriderm cream.

	Source of variation	Sum of squares	Degree of freedom	Mean square	F	F critical
TOL	Between Groups	7.90	2.00	3.95	3.40	5.14
	Within Groups	6.97	6.00	1.16		
	Total	14.87	8.00			
BETA	Between Groups	2.81	2.00	1.40	2.41	
	Within Groups	3.50	6.00	0.58		
	Total	6.31	8.00			
CC	Between Groups	0.45	2.00	0.22	0.47	
	Within Groups	2.85	6.00	0.47		
	Total	3.30	8.00			

## AI-driven sustainability assessment using the MA tool

This study presents UV-spectrophotometric procedures for analysis of pharmaceutical cream formulations. The analytical workflow comprises two main stages: ex situ macro extraction (sample preparation) and manual UV measurement. Due to the semisolid matrix, sample preparation constitutes the primary sustainability hotspot and is expected to dominate reagent/solvent use and waste generation relative to the measurement step.

To obtain a structured and quantitative sustainability profile, the method was evaluated using the MA Tool (2025)^22^, freely accessible web-based platform that integrates four complementary assessment domains into a single 51-question protocol: GEMAM (Green Experimental Matrix Assessment), BAGI (Blue Applicability Grade Index), RAPI (Red Analytical Performance Index), and VIGI (Violet Innovation Grade Index)^23–27^. The MA Tool applies predefined, rule driven scoring criteria to user provided method information and returns domain scores and a composite Whiteness Score. The platform requires no installation and performs calculations client-side, supporting transparent scoring and data privacy.

### Copilot assistance and system boundary

To reduce the time required for completing the 51-item MA Tool assessment (particularly compiling and calculating RAPI-related parameters), Microsoft Copilot (web) was used as an external assistant. Copilot was provided with the MA Tool documentation and the method-specific inputs required for scoring. Copilot was used to structure inputs and draft scores/diagram; no model fine-tuning/training was performed. Copilot outputs were treated as drafts and were verified item-by-item against the published MA Tool criteria. The Copilot-generated scores matched the manually verified results, while reducing the time required to compile scoring tables and diagrams^28^.

The sustainability assessment was bounded to the analytical method workflow (chemicals/solvents, waste generation/treatment assumptions, energy demand, instrument operation, throughput, and validation performance).

### MA tool results and interpretation

**Fig. 7:**
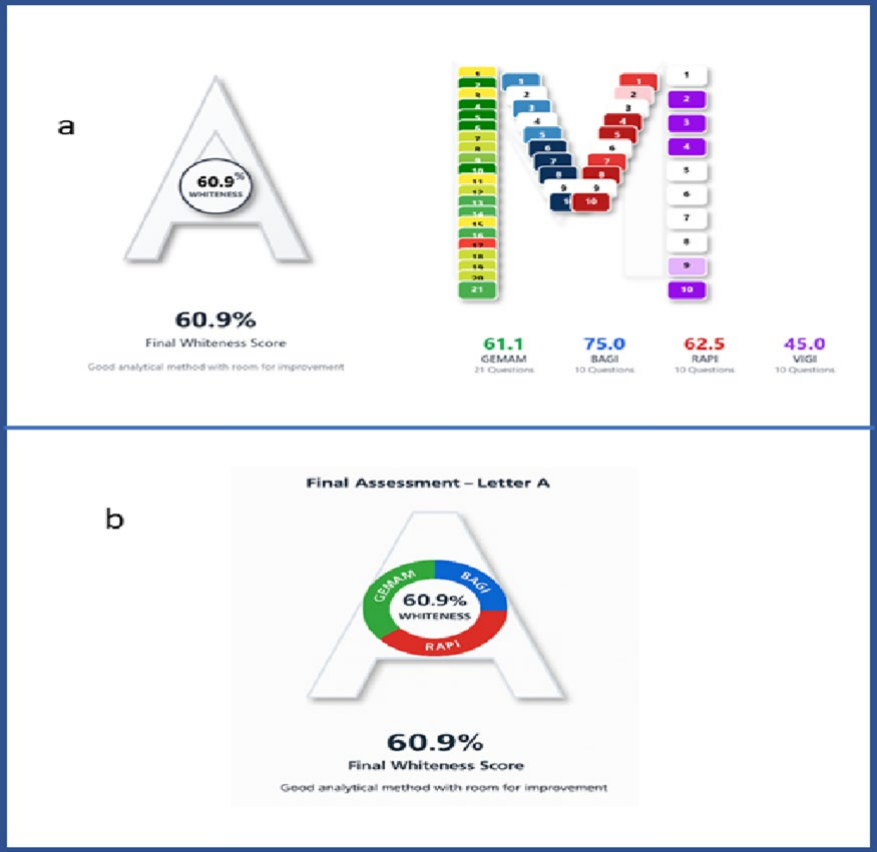
Comparison of manual and AI-assisted MA Tool outputs for the developed UV method. (**a**) Manually generated MA Tool “A” and “M” diagrams. (**b**) Copilot-assisted “A” diagram.

The MA Tool evaluation yielded a final Whiteness Score of 60.9%. Domain-specific scores were GEMAM 61.1%, BAGI 75.0%, RAPI 62.5%, and VIGI 45.0%. Visual outputs included manually generated “A” and “M” diagrams using the standard web MA Tool workflow (Fig. 7a) and “A” diagram drafted with Copilot assistance (Fig. 7b).

As shown in Fig. 7a–b, the Copilot-assisted “A” diagram reproduces the manually generated MA Tool scores exactly (Whiteness 60.9% and identical domain scores), supporting that AI use was limited to time-saving drafting while the final scoring remained rule-based and manually verified.

The score profile is consistent with the method design: BAGI was relatively high due to procedural simplicity and feasibility for routine use, whereas GEMAM reflects the environmental burden of sample preparation in a cream matrix. The lower VIGI score is mainly attributable to limited automation/advanced instrumentation and limited implementation of formal AQbD/advanced optimization within the experimental workflow. Accordingly, future sustainability gains should primarily target the macro extraction step (solvent/reagent reduction and greener substitution where feasible) together with implementation of basic waste handling/treatment.

The Copilot prompt used in this work is provided in the Supplementary Information S3 to facilitate reproducibility and transparency.

The developed UV procedure offers a cost-effective^29^, environmentally responsible solution for drug analysis in laboratories with limited resources, while AI tools significantly enhance workflow efficiency, reproducibility, and scientific impact.

## Conclusion and future recommendation

This study presents an innovative, eco-friendly UV–Vis spectrophotometric framework for the accurate analysis of TOL, BETA, and CC following physical and mathematical separation of GEN and CLIO, integrating analyst-guided AI-assisted data handling using the free version of ChatGPT with conventional processing via Spectra Manager^®^ software. ChatGPT was effectively employed to generate calibration graphs, regression equations, and evaluate accuracy and precision, enabling simultaneous analysis of laboratory mixtures and pharmaceutical formulations in a single step with significant savings in time and effort. Guided by fundamental analytical principles, the AI–analyst interaction combines human expertise with machine intelligence to deliver reliable and reproducible results without reliance on proprietary software, thereby enhancing accessibility for resource-limited laboratories and facilitating global collaboration through shareable spectral data. Among the evaluated approaches, AUTO-DD-DD showed superior capability in resolving severely overlapping spectra compared to MAN-DD-DD, while DD-DDE improved sensitivity and robustness for TOL and CC, with automated divisor selection and wavelength optimization increasing adaptability to other multicomponent systems. In parallel, the AI-assisted MA Tool provided a transparent and comprehensive sustainability assessment across environmental, performance, applicability, and innovation domains, accelerating calculations, simplifying complex RAPI evaluation, and improving reproducibility. Overall, integrating AI into spectrophotometric analysis advances Smart Analytical Chemistry by enabling rapid, non-destructive, and high-throughput drug analysis, supporting standardized and collaborative workflows, strengthening sustainability assessment, and reducing dependence on costly solvents and instrumentation typically associated with HPLC, while preserving the essential role of chemist expertise in method development, validation, and interpretation.

## Supplementary Information

Below is the link to the electronic supplementary material.

Supplementary Material 1

## Data Availability

All data generated or analyzed during this study are included in this published article.

## Abbreviations

MAN-DD-DD: Manual double divisor derivative ratio methodviaSpectra Manager^®^Software
MAN-DD-DDE: Manual derivative ratio extraction using double divisor via Spectra Manger^®^ software
AUTO-DD-DD: Automated double divisor derivative ratio via AI tools
AI: Artificial intelligence
CP: Coincidence point

## Declarations

### AI assistance

The authors acknowledge the use of Microsoft Copilot (https://copilot.microsoft.com) and ChatGPT-5.1, AI-powered tools, which were employed exclusively to assist in method optimization and language refinement. All scientific judgments, interpretations, and conclusions presented in this manuscript are solely those of the authors.
