# Supplementary material for: Artificial intelligence versus traditional approaches in multicomponent spectral analysis
Source: Sci Rep. 2026 Mar 1;16:7835. doi: 10.1038/s41598-026-39433-3 (PMC12953902; doi:10.1038/s41598-026-39433-3)
Supplement: Supplementary file 1 — Supplementary Material 1 [file 41598_2026_39433_MOESM1_ESM.docx]

Supplementary data

**S 1**


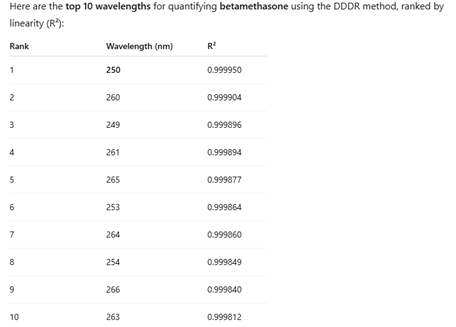


# **S2: Supplementary Information: Method Details for CLIO and GEN**

## **1. Gentamicin (GEN)**

**Instrumentation:**
Fluorescence measurements were performed using a Shimadzu RF-1501 spectrofluorimeter with a 1 cm quartz cell, low sensitivity, and 2.5 nm bandwidth.

**Reagents:**

- **Borate Buffer (pH 10.4):** Dissolve 2.473 g boric acid in 100 mL distilled water, adjust pH with 45% w/v KOH solution, then complete to 100 mL with water.
- **o-Phthalaldehyde (OPA) reagent:** Prepared according to BP method: dissolve 1.0 g OPA in 5 mL methanol, add 95 mL borate buffer, then add 2.0 mL mercaptoacetic acid (thioglycolic acid) and readjust pH to 10.4 with 50% w/v KOH solution.

**Linearity and Calibration:**
Portions equivalent to 0.25–1.25 µg/mL GEN were transferred from working solutions (25.0 µg/mL) into 10-mL volumetric flasks. Add 0.5 mL OPA reagent, complete to volume with methanol, heat in a water bath at 60 °C for 15 min, then cool. Excitation and emission spectra were scanned against a similarly prepared blank and stored on the computer. Calibration graphs were constructed, and regression equations were computed.

**Analysis in Laboratory Prepared Mixtures:**
Solvent was evaporated, and 50:50 (v/v) chloroform:water added. Shake in a separating funnel. Aliquots of 5.0 mL were transferred to 10-mL volumetric flasks, add 0.5 mL OPA reagent, complete to volume with methanol, heat at 60 °C for 15 min, then cool. Excitation at 359 nm and emission at 419 nm were measured against a blank.

**Validation Parameters:**

- **Linearity range:** 0.25–1.25 µg/mL
- **Limit of detection (LOD):** 0.01 µg/mL


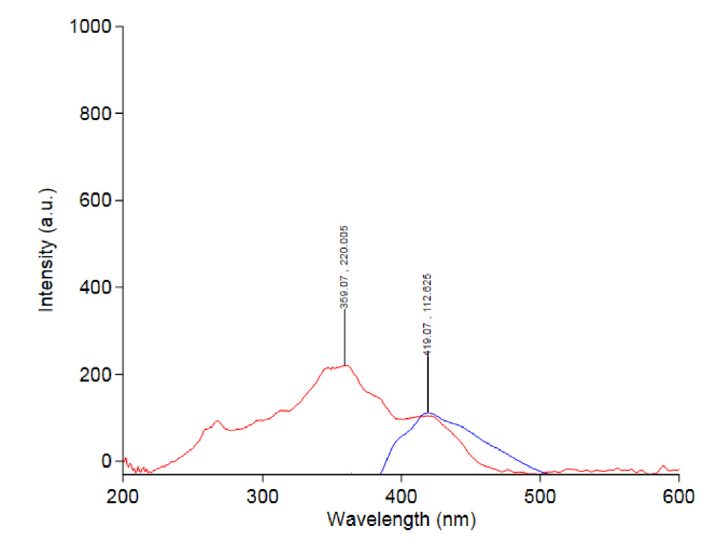


The excitation and emission parameters of GEN.

## **2. Clioquinol (CLIO)**

**Spectrophotometric Methods:**

**Constant multiplication (CM) Method:**
From the Zero-order absorption spectra (D₀) of CLIO, calibration graph was constructed by λmax at 254.8 versus the corresponding CLIO concentration to compute the regression equation.

**Validation Parameters:**

- **Linearity range:** 1–7 µg/mL
- **Limit of detection (LOD):** 0.07 µg/mL

The zero order absorption spectra of CLIO **:** 1–7 µg/mL

**S3 . AI‑assisted MA Tool workflow (Microsoft Copilot prompt and procedure)**

Microsoft Copilot (web) was prompted using a structured workflow and provided with (i) the MA Tool PDF document and (ii) our method‑specific data to support consistent scoring and diagram generation. This was done after uploading the original MA Tool article and the supporting pdf references describing the four MA Tool domains (GEMAM, BAGI, RAPI, and VIGI).

*"You are an expert assistant in green and smart analytical chemistry. Your task is to guide the user step-by-step in performing a comprehensive sustainability assessment of a UV-spectrophotometric method for pharmaceutical cream formulations using the MA Tool (2025). Follow these instructions carefully."*

***Step 1: Understand the Analytical Method***

- *Request full method specifications: sample type, matrix, extraction/preparation steps, instrumentation, validation parameters (repeatability, inter- and intra-day bias, recovery, LOQ, linearity, selectivity), throughput, waste, and energy consumption*

***Step 2: Explain the MA Tool Domains***

- ***GEMAM:*** *Sample preparation, reagent hazards, energy consumption, waste treatment*
- ***BAGI:*** *Technique versatility, throughput, reagent availability, sample volume, automation*
- ***RAPI:*** *Repeatability, bias (average of intra- and inter-day values across three analytes), recovery, LOQ, linearity, robustness, selectivity*
- ***VIGI:*** *AI integration, AQbD tools, novelty, automation, advanced instrumentation*

***Step 3: Clarify Scoring Logic***

- *Explain scoring thresholds and rules for all 51 questions****.***

***Step 4: Perform Evaluation***

- *Answer all 51 MA Tool questions*
- *Automatically generate RAPI scoring table, including average bias (%), LOQ (%), and working range*

***Step 5: Visual Representation***

- *Generate a clear* ***“A” diagram*** *showing domain performance and sustainability balance (as shown in* ***Figure*** *7, b)*

***Step 6: Recommendations***

- *Identify areas where scores are suboptimal*
- *Suggest actionable improvements*

***Step 7: Review and Validate***

- *Compare AI-generated outputs with manual scoring to ensure reliability, reproducibility, and transparency*
